# CD8^+^ T cell evasion mandates CD4^+^ T cell control of chronic gamma-herpesvirus infection

**DOI:** 10.1371/journal.ppat.1006311

**Published:** 2017-04-10

**Authors:** Cindy S. E. Tan, Clara Lawler, Philip G. Stevenson

**Affiliations:** School of Chemistry and Molecular Biosciences, University of Queensland and Royal Children’s Hospital, Brisbane, Australia; La Jolla Institute for Allergy and Immunology, UNITED STATES

## Abstract

Gamma-herpesvirus infections are regulated by both CD4^+^ and CD8^+^ T cells. However clinical disease occurs mainly in CD4^+^ T cell-deficient hosts. In CD4^+^ T cell-deficient mice, CD8^+^ T cells control acute but not chronic lung infection by Murid Herpesvirus-4 (MuHV-4). We show that acute and chronic lung infections differ in distribution: most acute infection was epithelial, whereas most chronic infection was in myeloid cells. CD8^+^ T cells controlled epithelial infection, but CD4^+^ T cells and IFNγ were required to control myeloid cell infection. Disrupting the MuHV-4 K3, which degrades MHC class I heavy chains, increased viral epitope presentation by infected lung alveolar macrophages and allowed CD8^+^ T cells to prevent disease. Thus, viral CD8^+^ T cell evasion led to niche-specific immune control, and an essential role for CD4^+^ T cells in limiting chronic infection.

## Author summary

Gamma-herpesviruses chronically infect most people. While infection is usually asymptomatic, disease occurs if the immune system is weakened. Understanding how immune control normally works should provide a basis for preventing disease. In mice, CD8^+^ T cells can control acute gamma-herpesvirus infection but not chronic infection. We show that acute and chronic infections involve different cell types. CD8^+^ T cells controlled epithelial cell infection, which predominated acutely, but they could not control chronic macrophage infection unless viral immune evasion was disabled. Instead CD4^+^ T cells were required. Thus, viral evasion made host defence cell type-specific: CD8^+^ T cells controlled epithelial cell infection; CD4^+^ T cells controlled macrophage infection; and comprehensive control required both T cell subsets.

## Introduction

Herpesviruses chronically infect immunocompetent hosts. CD4^+^ and CD8^+^ T cells both help to contain these infections, but disease occurs mainly when CD4^+^ T cells are lacking [1], implying that they have particular importance. Among the gamma-herpesviruses, CD4^+^ T cell deficiency leads Epstein-Barr virus (EBV) to cause lymphoproliferative disease and oral hairy leukoplakia, a virus-productive epithelial lesion [2]; it leads the Kaposi's Sarcoma-associated Herpesvirus (KSHV) to cause endothelial cell proliferation with inflammation and viral lytic gene expression [3]; and it leads MuHV-4 to replicate chronically in the lungs [4]. Thus the pathologies of CD4^+^ T cell-deficient hosts vary, but increased lytic infection is a common theme.

Gamma-herpesviruses characteristically persist in lymphocytes. EBV, KSHV and MuHV-4 all persist in B cells. However to reach B cells then re-emerge to reach new hosts they must also infect other cell types. EBV emerging from plasma cells [5] reaches the saliva via epithelial cells [6]. The normal association of plasma cells with mucosal epithelial cells provides a basis for virus transfer. How EBV reaches naive B cells is less well understood, as they have little direct communication with mucosal epithelia. Antigen presentation by myeloid cells provides a potential route to naive B cells. KSHV can infect many cell types [7], including myeloid cells [8]; EBV colonization of NK cell and T cell cancers [9] suggests a broader tropism than is usually evident *in vitro*; and MuHV-4, after epithelial host entry [10, 11], reaches B cells via dendritic cells [12]. Myeloid cell infection also features prominently in acute MuHV-4 colonization of splenic B cells [13–15]. Thus while only modestly efficient *in vitro* [16] and hard to detect in the long-term [13], myeloid cell infection plays a key role in MuHV-4 tropism.

Acute MuHV-4 lung infection is controlled mainly by CD8^+^ T cells [17]. They also help to control splenic B cell infection [18], and macrophage infection after peritoneal challenge [19]. β_2_-microglobulin-deficient BALB/c mice show a 3-fold increase in lymphoma incidence after MuHV-4 infection [20]. However β_2_-microglobulin deficiency impairs more than just than CD8^+^ T cell function, for example it reduces serum IgG [21]. Therefore the increased lymphoma incidence was not just CD8^+^ T cell dependent. Moreover few if any lymphoma cells showed evidence of MuHV-4 infection [20], and no lymphomas were seen in MuHV-4-infected β_2_M^-/-^ C57BL/6 [22] or 129 mice [20]. Inbred mice are prone to lymphomagenesis by strain-polymorphic endogenous retroviruses [23], and gamma-herpesviruses can transactivate retroviruses [24]. Therefore the ontogeny of the lymphomas remains unclear. The most obvious consequence of CD8^+^ T cell deficiency for MuHV-4 is increased lytic infection [22]. While cancers are the most harmful outcome of EBV infection, T cell deficiency again mainly increases lytic infection [25].

CD4^+^ T cell-deficient mice also show more lytic infection. However unlike CD8^+^ T cell-deficient mice, and despite maintaining strong anti-viral CD8^+^ T cell responses [26–28], they suffer a wasting disease [4]. Anti-MuHV-4 antibody responses help to contain infection [29] and depend on CD4^+^ T cells [30], but a lack of antibody alone does not explain the disease of CD4^+^ T cell-deficient mice, as B cell-deficient mice survive [31]. Acutely CD4^+^ T cells suppress MuHV-4 replication independently of B cells, with an important role for interferon-γ (IFNγ) [32, 33], so their effector function may also be important for long-term MuHV-4 control.

Why CD8^+^ T cells alone fail to control MuHV-4 is important to understand because they are a therapeutic focus for EBV. CD8^+^ T cell evasion is a near universal characteristic of mammalian herpesviruses. While its molecular mechanisms have been studied extensively, its impact on infection is less well understood. The MuHV-4 K3 degrades MHC class I (MHC I) [34] and the transporter associated with antigen processing (TAP) [35]. K3 disruption impairs virus-driven lymphoproliferation [36]. We show that in chronic infection, K3 protects lung macrophages against CD8^+^ T cells and so makes CD4^+^ T cells essential to prevent disease.

## Results

### Different cell types support acute and chronic MuHV-4 lung infections

**Fig 1:**
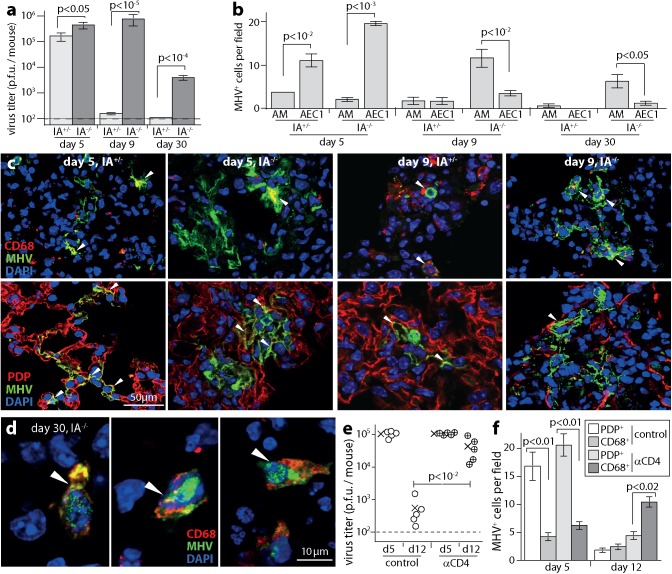
CD4^+^ T cell deficiency promotes chronic macrophage infection. **a.** IA^-/-^ mice or IA^+/-^ littermate controls were given MuHV-4 i.n. (10^4^ p.f.u.). Lungs were then plaque assayed for infectious virus. Bars = mean ± SEM of individuals (n = 6). Dashed line = assay sensitivity limit. **b.** Lung sections of mice infected as in **a** were stained for viral lytic antigens (MHV) and for AM (CD68) and AEC1 (PDP). MHV^+^ cells were counted across 3 fields of view per section for 3 sections of each of 3 mice. Bars = mean ± SEM. No viral antigen staining was seen in lungs of naive mice. **c.** Example images from **b** show MHV^+^CD68^+^ and MHV^+^PDP^+^ cells (arrows) at d9. Red / green co-localization appears as yellow. Each AEC1 has a large surface area and often a complex shape. Thus, cell counts were of nuclei within areas of PDP staining. See also S1 Fig Staining of naive lungs is shown in S2 Fig. **d.** Example images from **b** show MHV^+^ AM in IA^-/-^ mice at d30 (arrows). Lungs of IA^+/-^ mice showed no MHV antigen staining at this time. See also S3 Fig. **e.** C57BL/6 mice depleted of CD4^+^ T cells (αCD4) or not (control) were given MuHV-4 i.n. (10^4^ p.f.u.). Lungs were plaque assayed for infectious virus at d5 or d12. Circles = individual mice (5 per group), crosses = means, dashed line = assay sensitivity limit. **f**. Lung sections from **e** were stained for MHV and CD68 or PDP. Bars = mean ± SEM counts of MHV^+^ cells across 3 fields of view per section for 3 sections from each of 3 mice.

MuHV-4 replicates chronically in MHC class II (MHC II)-deficient (IA^-/-^) C57BL/6 mice, which lack classical CD4^+^ T cells [4]. Intranasal (i.n.) BAC-derived MuHV-4 reached similar peak titers in the lungs of IA^-/-^ and wild-type control mice (WT, IA^+/-^) at day (d) 5 post-infection, but then maintained higher titers in IA^-/-^ mice (Fig 1A). The main cell populations of the lung alveoli are type 1 epithelial cells (AEC1), which have a characteristically flattened shape with a large surface area for gas exchange, and express podoplanin (PDP); type 2 AEC (AEC2), which express surfactant proteins; and alveolar macrophages (AM), which phagocytose inhaled debris and express CD68. MuHV-4 entering the lungs binds to AEC1 and is then captured by AM [11]. Subsequent replication in AM allows spread back to AEC1 and makes them the main site of acute virus production. At d5, immunostaining for viral lytic antigens showed mainly AEC1 infection in both WT and IA^-/-^ mouse lungs (Fig 1B and 1C; S1 Fig; S2 Fig). By d9, WT lungs contained few infected cells. IA^-/-^ lungs contained significantly more (p<0.01), and most of these were AM. AM infection remained detectable at d30 in IA^-/-^ (Fig 1B and 1D; S3 Fig) but not WT mice. Few AEC2 or lung B cells expressed viral lytic antigens. Thus WT mice resolved acute AEC1 infection, while in IA^-/-^ mice it evolved into a chronic infection of AM.

CD4^+^ T cell-depleted C57BL/6 mice showed a similar picture (Fig 1E and 1F). The virus titers in depleted mouse lungs were equivalent to those of undepleted controls at d5, then higher at d12 (Fig 1E). Lung sections showed mainly AEC1 infection at d5, and mainly AM infection at d12 (Fig 1F). We did not see evidence of chronic epithelial cell infection, as reported for B cell-deficient mice [37]. Ongoing myeloid cell infection may seeds epithelial infection in some settings, but the main cell type supporting chronic lung infection in CD4^+^ T cell-deficient mice was myeloid.

### CD8^+^ T cells control AEC1 infection

**Fig 2:**
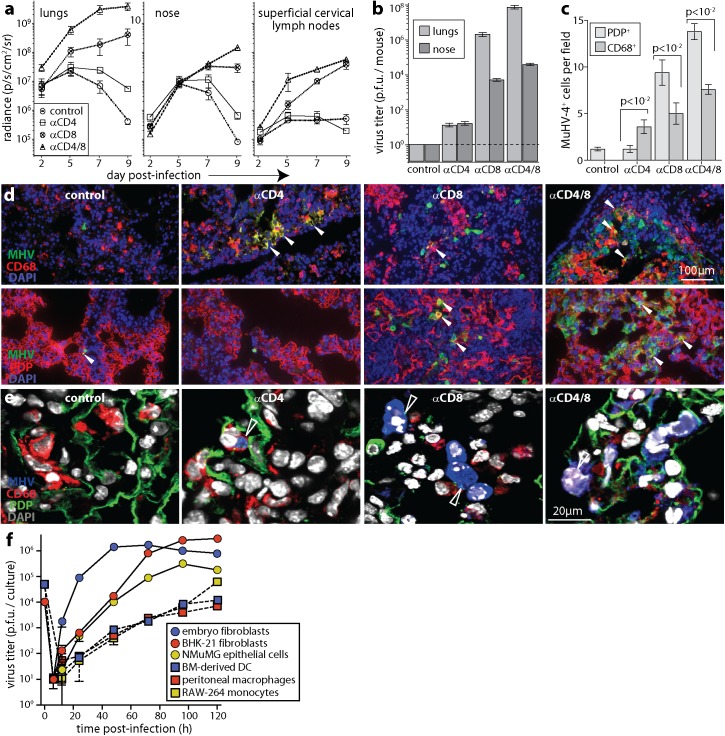
CD8^+^ T cells control AEC1 infection; CD4^+^ T cells control AM infection. **a.** BALB/c mice depleted of CD4^+^ T cells (αCD4), CD8^+^ T cells (αCD8), both (αCD4/8) or neither (control) were given MHV-LUC i.n. (10^4^ p.f.u.). Infection was tracked by live imaging of light emission. Mean ± SEM radiance is shown for 6 mice per group. D7 and d9 signals were significantly higher in depleted mice than controls (p<10^−2^), αCD8 was higher than αCD4 (p<10^−2^) and αCD4/8 was higher than αCD8 (p<10^−3^). Noses at d9 showed the same hierarchy (p<10^−2^). SCLN signals were significantly higher in αCD8 and αCD4/8 mice than controls at d7 and d9 (p<10^−3^) and significantly lower in αCD4 mice at d9 (p<0.05). **b.** Lungs and noses of mice as in **a** were plaque assayed for infectious virus at d9. Bars show mean ± SEM of 6 mice per group. All depletions increased virus titers relative to controls (p<10^−4^), αCD8 gave a greater increase than αCD4 (p<10^−4^) and αCD4/8 gave a greater increase than αCD8 (p<10^−2^). **c.** BALB/c mice were treated as in **a**. 9d later lung sections were stained for MHV and CD68 or PDP. Bars = mean ± SEM counts for 3 mice, counting cells across 3 fields of view per section for 3 sections of each mouse. **d.** Example images from **c** show infected CD68^+^ and PDP^+^ cells. Red / green co-localization appears as yellow. **e.** Higher magnification, triple-stained images show a CD68^+^ cell with MHV antigen after anti-CD4, MHV^+^ cells with residual PDP staining after anti-CD8, and a MHV^+^CD68^+^ cell after anti-CD4/8 (arrows). Red+blue co-localization appears as pink; green+blue co-localization appears as cyan. **f.** NMuMG epithelial cells, BHK-21 fibroblasts and mouse embryo-derived fibroblasts were infected with MuHV-4 at 0.01 p.f.u. / cell; RAW-264 monocytes, peritoneal macrophages and bone marrow-derived dendritic cells were infected at 0.1 p.f.u. / cell. After 4h the cells were washed in pH = 3 buffer to inactivate residual input virions. Infection was then tracked by plaque assay of replicate cultures. Mean ± SD of triplicate cultures are shown. Despite higher inocula, myeloid cells consistently yielded less virus from 24h onwards (p<10^−2^ by Student’s 2-tailed unpaired t test, comparing myeloid culture titers at each time point with non-myeloid cultures).

MuHV-4 causes disease more readily in BALB/c than in C57BL/6 mice [20], with acute protection being CD8^+^ T cell-dependent [17]. We tested whether BALB/c mice also showed CD4^+^ T cell-dependent myeloid infection control (Fig 2). Live imaging of i.n. luciferase^+^ MuHV-4 showed CD4^+^ T cell depletion significantly increasing lung and nose infections at d7 and d9 (Fig 2A). CD8^+^ T cell depletion had significantly more effect, and dual depletion had more effect still. CD8^+^ T cell depletion also increased colonization of the superficial cervical lymph nodes (SCLN), which drain the upper respiratory tract, while CD4^+^ T cell depletion reduced SCLN colonization, consistent with the amplification of B cell infection in lymphoid tissue being CD4^+^ T cell-dependent [38]. D9 virus titers in lungs and noses (Fig 2B) matched the luciferase signals, with dual depleted > CD8^+^ T cell depleted > CD4^+^ T cell depleted > undepleted controls. Thus acutely, when epithelial infection predominated, CD8^+^ T cells contributed more than CD4^+^ T cells to controlling virus replication in both the upper and lower respiratory tract.

Immunostaining infected BALB/c lungs at d9 (Fig 2C–2E) showed significantly more AEC1 than AM infection in all groups except that depleted of CD4^+^ T cells, which showed significantly more AM infection. Epithelial and fibroblast infections were consistently more virus-productive than myeloid cell infection *in vitro* (Fig 2F), and the higher virus titers of mice with more AEC1 infection were consistent with AEC1 producing more virus acutely than AM. Thus, CD8^+^ T cells appeared to be more important than CD4^+^ T cells for acute infection control because they targeted a more immediately virus-productive cell type—AEC1—while a lack of CD4^+^ T cells increased AM infection.

### CD4^+^ T cells and IFNγ control MuHV-4 replication in the lungs

**Fig 3:**
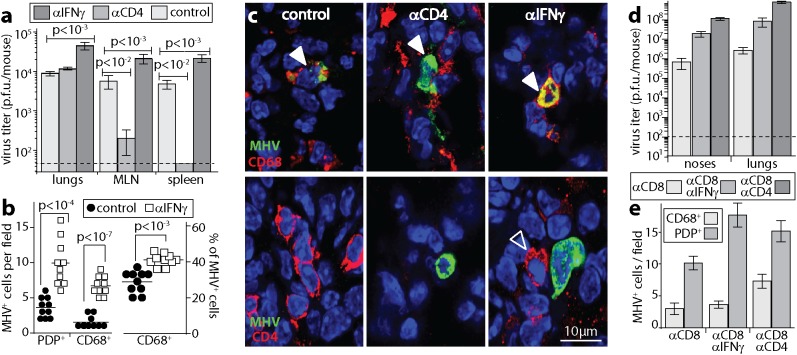
IFNγ neutralization also increases AM infection. **a.** C57BL/6 mice given IFNγ neutralizing antibody (αIFNγ), depleted of CD4^+^ T cells (αCD4) or left untreated (control), were given MuHV-4 i.n. (10^4^ p.f.u.). 9d later lungs were plaque assayed for infectious virus, and mediastinal LN (MLN) and spleens infectious center assayed for latent virus. Bars = mean ± SEM of 6 mice per group. Dashed line = assay sensitivity limit. **b.** Mice were treated as in **a**. 9d later lung sections were stained for viral lytic antigens (MHV) plus CD68, PDP or CD4. Quantitation of staining showed that αIFNγ mice had significantly more MHV^+^ AEC1 and AM than controls, with AM accounting for a significantly greater proportion of all MHV^+^ cells. **c.** Examples of staining as quantitated in **b**. Nuclei were stained with DAPI. Filled arrows show example MHV^+^ AM. Red / green co-localization appears as yellow. The open arrow shows a CD4^+^ T cell adjacent to an infected cell. This was commonly observed after IFNγ neutralization. **d.** Mice were depleted of CD8^+^ T cells (αCD8), given also IFNγ neutralizing (αCD8αIFNγ) or CD4^+^ T cell depleting antibodies (αCD8αCD4), and all mice were then infected with MuHV-4 i.n. (10^4^ p.f.u.). 9d later infectious virus was plaque assayed in noses and lungs. Bars = mean ± SEM of 6 mice per group. Dashed line = assay sensitivity limit. αCD8αIFNγ significantly increased titers above αCD8 and αCD8αCD4 significantly increased titers above αCD8αIFNγ (p<0.01). **e.** Lung sections of mice treated as in **d** were stained for MHV and cell type markers (CD68, PDP). Cells were counted for 3 fields of view per section, across 3 sections each of 3 mice per group. Bars = mean ± SEM counts of individual mice. αCD4αCD8 significantly increased CD68^+^ infected cell numbers over αCD8 alone (p<0.05). αIFNγ/αCD8 did not (p>0.2).

In mice lacking B cells and CD8^+^ T cells, IFNγ is required for acute infection control [32, 33], suggesting that it mediates the anti-viral effect of CD4^+^ T cells. It also inhibits *ex vivo* MuHV-4 reactivation from peritoneal macrophages [39]. In otherwise immunocompetent BALB/c mice, IFNγ neutralization increased d9 lung virus titers significantly more than did CD4^+^ T cell depletion (Fig 3A), and increased both AM and AEC1 infections (Fig 3B and 3C), implying that it also mediated other anti-viral effects. Again CD4^+^ T cell depletion decreased MuHV-4 colonization of lymphoid tissue, whereas IFNγ neutralization increased it (Fig 3A).

**Fig 4:**
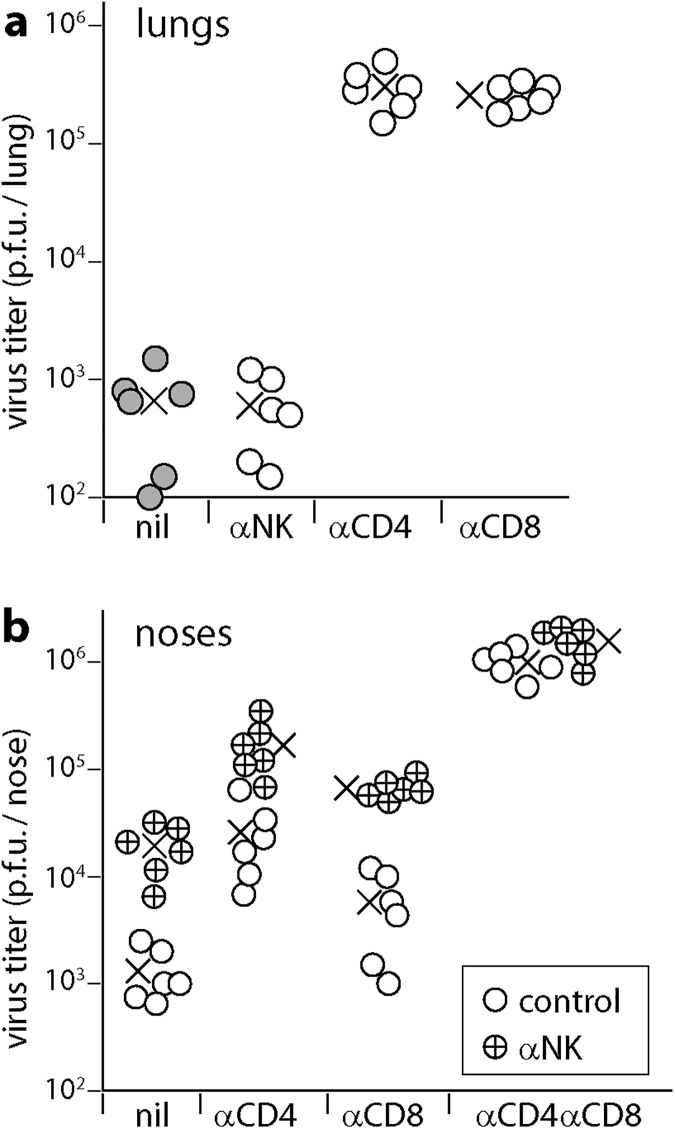
NK cell depletion increases MuHV-4 infection in noses but not in lungs. **a.** Mice were depleted of NK cells (αNK), CD4^+^ T cells (αCD4) or CD8^+^ T cells (αCD8), or left undepleted (nil), then infected i.n. with MuHV-4 (10^4^ p.f.u.). 10 days later, lungs were plaque assayed for infectious virus. Circles show individual mice, crosses shows means. The x-axis shows the lower limit of assay sensitivity. αCD4 and αCD8 significantly increased virus titers above the undepleted controls (p<10^−5^). αNK did not (p>0.5). **b.** Mice were depleted of CD4^+^ T cells (αCD4), CD8^+^ T cells (αCD8), both (αCD4αCD8) or neither (nil). Half of each group was then depleted of NK cells (αNK) or not (control). All mice were then given MuHV-4 i.n. (10^5^ p.f.u. without anaesthesia). 10 days later, noses were plaque assayed for infectious virus. Circles show individuals, crosses shows means. The x-axis shows the lower limit of assay sensitivity.

CD4^+^ T cells, CD8^+^ T cells and NK cells all produce IFNγ. When CD8^+^ T cells were eliminated, CD4^+^ T cell depletion increased virus titers and AM infection significantly more than did IFNγ neutralization (Fig 3D and 3E). Therefore while IFNγ was an important CD4^+^ T cell-mediated defence, it was not the only one and it contributed also to CD8^+^ T cell-mediated defence. NK cell depletion does not affect the course of MuHV-4 lung infection in otherwise immunocompetent mice [40], but increases LN infection by MuHV-4 inoculated into footpads [41]. In C57BL/6 mice NK cells did not make a significant contribution to infection control in lungs at d10, making it unlikely that they were a significant source of IFNγ in this setting (Fig 4A). They did contribute to infection control in noses (Fig 4B). Here NK cell depletion increased virus titers regardless of whether CD4^+^ T cells were depleted. Therefore CD4^+^ T cells and NK cells functioned as independent defences.

### CD4^+^ T cells control MuHV-4 replication in MHC II^+^ cells

**Fig 5:**
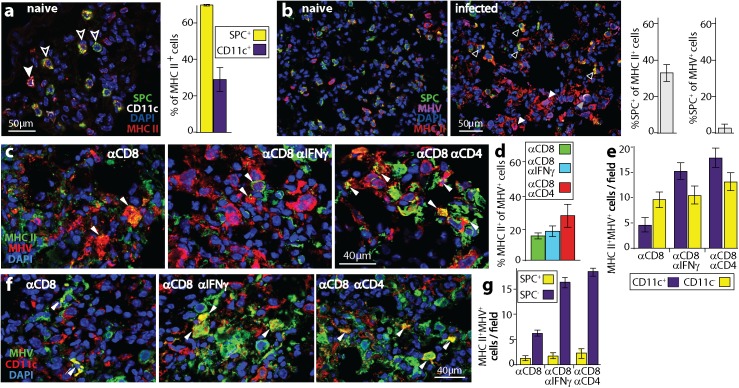
A lack of CD4^+^ T cells or of IFNγ increases MHC II^+^ cell infection. **a.** Naive mouse lungs were stained for AEC2 (SPC^+^), AM (CD11c^+^), and MHC II. The filled arrow shows an MHC II^+^ AM. White / red co-localization appears as pink. Most MHC II^+^ cells were AEC2 (open arrows). Red / green co-localization appears as yellow. Bars show mean ± SEM of counts for 3 fields of view per section, for 2 sections from each of 2 mice. **b.** Naive and d5 infected lungs were stained for SPC, MHC II and viral antigens (MHV). In infected lungs, open arrows show MHC II^+^ AEC2, which are uninfected (MHV^-^); white filled arrows show infected MHC II^+^ cells, which are SPC^-^; the grey filled arrow shows a rare MHV^+^ AEC2 (colocalization appears as white), which is MHC II^-^. Fig 5a shows that in naive mice 70% of MHC II^+^ lung cells were SPC^+^. After infection, significantly fewer were SPC^+^ (p<0.001 by Student's 2 tailed unpaired t test), and negligible numbers of MHV^+^MHC II^+^ cells (<5%) were SPC^+^. Bars show mean ± SEM of counts for 3 fields of view per section, across 3 sections from each of 2 mice. **c.** Mice were depleted of CD8^+^ T cells (αCD8), given optionally either IFNγ neutralizing (αCD8αIFNγ) or CD4^+^ T cell depleting antibodies (αCD8αCD4), then given MuHV-4 i.n. (10^4^ p.f.u.). At d9 lungs were stained for MHV and MHC II. Arrows show dual positive cells. **d.** For samples as illustrated in **c**, cells were counted for 3 fields of view per section, across 3 sections from each of 3 mice per group. The proportion of MHV^+^ cells that were MHC II^+^ was significantly increased by αCD4 (p<0.05) but not by αIFNγ (p>0.2). **e.** MHC II^+^MHV^+^ cells of mice treated as in **b** were identified as CD11c^+^ or CD11c^-^. Cells were counted for 3 fields of view per section, across 3 sections from each of 3 mice per group. αIFNγ significantly increased MHV^+^MHC II^+^CD11c^+^ (p<0.01) but not MHV^+^MHC II^+^CD11c^-^ cell numbers (p>0.3). αCD4 significantly increased both (p<0.01, p<0.05). **f.** Example images are shown for the samples counted in **e**. Arrows show MHV^+^CD11c^+^ cells. **g.** Staining the same samples for SPC showed that essentially no MHV^+^MHC II^+^CD11c^-^ cells were AEC2.

CD4^+^ and CD8^+^ T cells differ in both target cell recognition and predominant effector functions: CD8^+^ act mostly via perforin and granzymes, while IFNγ is a key effector for CD4^+^ T cells [42]. Thus, CD4^+^ T cell-dependent myeloid infection control could have reflected either that only CD4^+^ T cells efficiently recognized infected myeloid cells (via MHC II), or that only IFNγ was able to control their infection. To explore these possibilities we tracked the infection of MHC II^+^ lung cells (Fig 5). In naive lungs, 1/3 of MHC II^+^ cells were CD11c^+^ AM or dendritic cells, and 2/3 were surfactant protein C precursor (SPC)^+^ AEC2 (Fig 5A). After MuHV-4 infection, most MHC II^+^ cells (>70%) were SPC^-^, presumably reflecting myeloid cell recruitment and MHC II up-regulation. Almost all MuHV-4^+^ cells (>95%) were SPC^-^, that is myeloid rather than AEC2 (Fig 5B).

Again we depleted CD8^+^ T cells as a source of IFNγ, then compared additional CD4^+^ T cell depletion with IFNγ neutralization. CD4^+^ T cell depletion increased the number of infected MHC II^+^ lung cells, while IFNγ neutralization gave only a non-significant increase (Fig 5C and 5D). Most AM express CD11c [43, 44]. Both IFNγ neutralization and CD4^+^ T cell depletion increased significantly the number of CD11c^+^MHC II^+^ infected cells. CD4^+^ T cell depletion but not IFNγ neutralization significantly increased the number of CD11c^-^MHC II^+^ infected cells (Fig 5E and 5F). SPC^+^ infection remained rare (Fig 5G), so CD11c^-^MHC II^+^MHV^+^ cells were presumably infected CD11c^-^ AM or infiltrating monocytes. Thus, CD4^+^ T cell depletion increased MuHV-4 infection of MHC II^+^ lung myeloid cells, and IFNγ neutralization reproduced much of this effect, consistent with IFNγ production being an important CD4^+^ T cell effector function. However the greater effect of CD4^+^ T cell depletion than IFNγ neutralization on lung myeloid cell infection implied that target cell recognition was the key parameter, rather than susceptibility to IFNγ.

### The MuHV-4 K3 prevents AM infection control by CD8^+^ T cells

**Fig 6:**
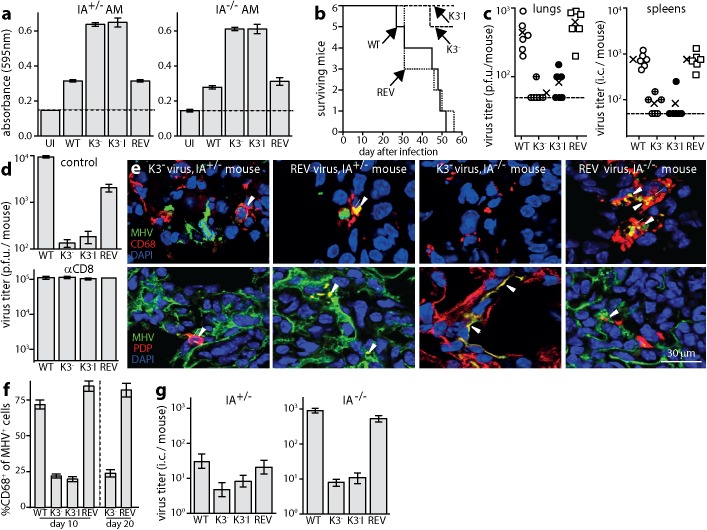
Chronic AM infection depends on viral CD8^+^ T cell evasion. **a.** AM from naive IA^+/-^ or IA^-/-^ mice were left uninfected (UI) or infected with wild-type (WT), K3 mutant (K3^-^), independent K3 mutant (K3^-^I) or K3^+^ revertant (REV) viruses. The cells were incubated overnight with a viral epitope-specific, β-gal^+^ T cell hybridoma. Hybridoma responses were detected by incubation with CPRG. Bars = mean ± SEM of triplicate cultures. K3^-^ viruses induced significantly greater responses than K3^+^ (p<10^−4^). Dashed lines = assay sensitivity limits. **b.** Groups of 6 IA^-/-^ mice were infected i.n. with K3^+^ and K3^-^ viruses as in **a** (10^4^ p.f.u.). Any losing >20% of their initial weight were euthanized. K3^-^ and K3^-^I caused significantly less disease than WT (p<10^−4^ by log rank test) whereas REV caused the same (p>0.7). **c.** IA^-/-^ mice were infected as in **b**. At d40 lungs were plaque assayed for infectious virus and spleens were infectious center (i.c.) assayed for infectious plus recoverable latent virus. Crosses = group means, other symbols = individual mice (5 per group). Dashed lines = sensitivity limits. K3^+^ virus titers were significantly higher than K3^-^ in both sites (p<0.01). **d.** IA^-/-^ mice were depleted or not of CD8^+^ T cells, then infected as in **b**. After 15d, lungs were plaque-assayed for infectious virus. Bars = mean ± SEM of 5 mice per group. In the control mice, K3^-^ virus titers were significantly less than K3^+^ (p<10^−3^); in αCD8 mice they were the same (p>0.9). **e.** Groups of 3 IA^-/-^ mice were infected as in **b**. 10d later, lung sections were stained for viral antigens (MHV), CD68 and PDP. Representative images are shown for K3^-^ and REV infections. Arrows show example dual positive cells. **f.** Immunostaining as illustrated in **e** was quantitated across 3 fields view for each of 3 sections for 3 mice per group. At both d10 and d20, areas of infection with K3^-^ viruses showed a significantly lower proportion of infected AM (CD68^+^) than with K3^+^ (p<10^−2^). **g.** Mice were infected as in **b**. At d10 AM recovered by bronchiolaveolar lavage were i.c. assayed for recoverable virus. In IA^-/-^ mice, K3^-^ viruses yielded significantly less AM infection than K3^+^ (p<10^−3^). K3^-^ and K3^-^I MuHV-4 also yielded less AM infection than WT in IA^+/-^ mice (p<0.03), and K3^-^ (but not K3^-^I) yielded significantly less than REV (p<0.04). Bars show mean ± SEM titers of 6 mice per group.

The importance of CD4^+^ T cell recognition for AM infection control implied poor CD8^+^ T cell recognition. Virus-specific CD8^+^ T cells were evidently functional in IA^-/-^ mice, as they controlled AEC1 infection; and myeloid cells are normally good CD8^+^ T cell targets [45]. However the MuHV-4 K3 degrades MHC I and TAP. To test whether K3 compromised CD8^+^ T cell recognition of AM, we exposed AM to K3^+^ or K3^-^ viruses, then measured epitope presentation to a MuHV-4-specific CD8^+^ T cell hybridoma (Fig 6A). K3 disruption significantly increased hybridoma stimulation by both WT and IA^-/-^ AM.

To establish whether better CD8^+^ T cell recognition of infected AM translated into better infection control, we infected IA^-/-^ mice with K3^+^ or K3^-^ MuHV-4. K3^+^ viruses caused significantly more disease (weight loss and general ill health requiring euthanasia) (Fig 6B) and reached higher titers in both lungs and spleens (Fig 6C). When CD8^+^ T cells were depleted, K3^+^ and K3^-^ viruses reached equivalent titers (Fig 6D). Immunostaining IA^-/-^ lung sections at infection d10 (Fig 6E and 6F) showed that K3^-^ viruses lacked the chronic AM infection of WT MuHV-4. Infectious center assays of AM recovered by lung washout (Fig 6G) confirmed greater infection by K3^+^ viruses. Therefore K3 limited CD8^+^ T cell-mediated control of MuHV-4 replication in lung myeloid cells.

## Discussion

Gamma-herpesviruses establish chronic, low-level transmission with generally few symptoms. Immunodeficiencies shift this equilibrium towards greater viral replication and disease. The key parameters of disease control in humans have been hard to define. Thus, anti-viral therapies have remained largely empirical. Our analysis of murine infection showed cell type-specific immune control. In mice lacking CD4^+^ T cells, CD8^+^ T cells still controlled epithelial infection. We did not see extensive B cell infection, presumably due to a lack of CD4^+^ T cell-dependent B cell proliferation [38]. However myeloid cell colonization, which is normally transient, became chronic and caused disease. This disease depended on CD8^+^ T cell evasion by the MuHV-4 K3: when K3 was disrupted, CD8^+^ T cells achieved long-term infection control and CD4^+^ T cells were not required.

Why did K3 protect infected macrophages and not epithelial cells against CD8^+^ T cells? K3 stabilization by tapasin [46] titrates its expression to the cellular capacity for antigen presentation, for example overcoming induction by IFNγ [35]. However immune evasion only raises the threshold for epitope presentation: peptides competing strongly for the few remaining MHC I complexes can still be recognized. Infected epithelial cells produced more virus than infected myeloid cells, implying that they produced more viral peptides, making break-through viral epitope presentation more likely. Cell type differences in susceptibility to CD8^+^ T cell attack are also possible. The faster clearance of pro-lytic MuHV-4 mutants from the lungs despite faster spread *in vitro* [47, 48] suggests that more indolent gamma-herpesvirus infections generally constitute more difficult immune targets. Myeloid cell infection is a common characteristic of lymphotropic viruses [49, 50], and MuHV-4 myeloid cell infection caused chronic disease despite limited virus production. Therefore poorly lytic infection should not exclude myeloid cell infection from consideration as a source of human gamma-herpesvirus-driven disease.

While IA^-/-^ mice make large CD8^+^ T cell responses to MuHV-4 [26], the non-uniformity of *in vivo* infection means that large immune responses are not always the most effective responses. For example MuHV-4-infected mice normally mount a large CD8^+^ T cell response to viral reactivation from latency in B cells [51]; yet if MHC I epitope presentation is enforced during viral episome maintenance [52], a relatively small CD8^+^ T cell response essentially abolishes latent B cell infection and hence also reactivation. The large acute CD8^+^ T cell responses to EBV lytic antigens [53] analogously imply a failure to suppress virus-driven lymphoproliferation. In IA^-/-^ mice CD8^+^ T cells kept AEC1 infection in check, but they did not shut down virus production by K3-protected myeloid cells. This required CD4^+^ T cells. Thus without CD4^+^ T cells, myeloid infection could constantly re-seed epithelial infection.

CD4^+^ T cells may be difficult for MuHV-4 to evade because it needs them to drive infected B cell proliferation. Also MHC II presents mainly exogenous antigens, so the presenting cells need not be infected, making them difficult to target. While CD4^+^ T cells have limited cytotoxic capacity, they can trigger apoptosis via tumor necrosis factor receptors and fas, activate myeloid cells to reduce their susceptibility to infection [54], and through cytokine signalling repress viral lytic gene expression directly [55]. Thus, there are abundant opportunities for CD4^+^ effector T cells to restrict MuHV-4 replication.

Most studies of anti-viral immunity have averaged outcomes across whole organs. Direct visualization is revealing additional complexity. For example CD8^+^ T cells combat cutaneous vaccinia virus by killing infected monocytes rather than keratinocytes [56]. Direct visualization revealed that immune evasion makes MuHV-4 control cell type-specific: CD8^+^ T cells controlled epithelial infection, and CD4^+^ T cells controlled myeloid infection. Thus CD4^+^ and CD8^+^ T cells co-operated, but less through classical help than through recognizing distinct components of a complex infection. Such niche-specific immune function suggests that single component vaccines eliciting mainly one effector class might only ever have partial efficacy against complex viruses; multi-component vaccines that prime complementary defences may be necessary for full protection.

## Materials and methods

### Mice

Adult C57BL/6, BALB/c, and C57BL/6 back-crossed IA^-/-^ mice [57] were infected i.n. with MuHV-4 (10^4^ p.f.u.) under isoflurane anaesthesia. Luciferase^+^ infection was imaged by peritoneal (i.p.) injection of D-luciferin (2mg/mouse, Pure Science) and charge-coupled camera scanning (IVIS spectrum, Xenogen). IFNγ was neutralized by i.p. mAb XMG1.2 (200μg/mouse/48h). CD4^+^ and CD8^+^ T cells were depleted by i.p. mAbs GK1.5 and 2.43 (200μg/mouse/48h, from 96h before infection). NK cells were depleted with NK1.1-specific mAb PK-136 (200μg/mouse/48h, from 48h before infection). Antibodies were from Bio X Cell. T cell subset depletion, measured by flow cytometry of spleen cells with antibodies to a distinct CD4 epitope (rat mAb RM4-4), and to CD8β (rat mAb H35-17.2) and was >95% complete. CD4^+^ T cell-depleted mice further lacked detectable MuHV-4-specific serum IgG by ELISA (S4 Fig). NK cell depletion, monitored by flow cytometric staining of spleen cells for CD49d with mAb DX5, was >90% complete. Statistical comparisons were by Student's 2-tailed unpaired t test unless otherwise stated.

### Ethics statement

All experiments were approved by the University of Queensland Animal Ethics Committee in accordance with the Australian code for the care and use of animals for scientific purposes, from the Australian National Health and Medical Research Council (project 301/13).

### Cells

Peritoneal macrophages were recovered by peritoneal lavage. After discarding non-adherent cells, the remainder were >80% F4/80^+^ by flow cytometry. Lung macrophages were recovered by bronchio-alveolar lavage and were >70% CD11c^+^ by flow cytometry. These cells, BHK-21 fibroblasts (American Type Culture Collection (ATCC) CCL-10), RAW-264 monocytes (ATCC TIB-71), NMuMG epithelial cells (ATCC CRL-1636), the 49100.2 T cell hybridoma [58], and mouse embryo fibroblasts were grown in Dulbecco’s Modified Eagle’s Medium with 2mM glutamine, 100IU/ml penicillin, 100μg/ml streptomycin, and 10% fetal calf serum (complete medium).

### Viruses

Luciferase^+^ [59] and GFP^+^ [60] MuHV-4, a K3^-^ mutant and its revertant [36] and an independent K3 mutant (K3^-^I) [54] were propagated in BHK-21 cells. Infected cell supernatants were cleared of debris by low speed centrifugation (200 x *g*, 5 min). Cell-free virions were then concentrated by ultracentrifugation (35,000 x *g*, 1.5h). To titer infectious virus, freeze-thawed samples were plated on BHK-21 cells (plaque assay); to titer total reactivatable MuHV-4, live cells were plated (infectious center assay) [60]. After 2h the cells were overlaid with complete medium plus 0.3% carboxymethylcellulose, cultured for 4d (37°C in complete medium) then fixed with 1% formaldehyde and stained with 0.1% toluidine blue.

### Immunostaining

Organs were fixed in 1% formaldehyde / 10mM sodium periodate / 75mM L-lysine (18h, 4°C), equilibrated in 30% sucrose (24h, 4°C), then frozen in OCT. 6μm sections were air-dried, washed 3x in PBS, blocked with 0.3% Triton X-100 / 5% donkey serum (1h, 23°C), then incubated (18h, 4°C) with combinations of antibodies to GFP (rabbit or goat pAb), CD68 (rat mAb, FA-11) (AbCam), B220 (rat mAb RA3-6B2), CD4 (rat mAb GK1.5), CD11c (hamster mAb N418, Abcam), MHC II (rat mAb M5/114, Serotec), SPC (goat pAb; Santa Cruz Biotechnology), podoplanin (goat pAb, R&D Systems), and MuHV-4 (rabbit pAb). The MuHV-4-immune serum was raised by 2x subcutaneous inoculation of rabbits with MuHV-4 virions (10^9^ p.f.u.). Like previously described immune sera [61] it recognizes a range of virion proteins by Western blot, including the products of ORF4 (gp70), M7 (gp150) and ORF65 (p20). Sections were washed 3×, incubated (1h, 23°C) with combinations of Alexa568-donkey anti-rat IgG pAb, Alexa488 or Alexa647-donkey anti rabbit IgG pAb, Alexa 647-goat anti-hamster IgG pAb (AbCam), and Alexa488-donkey anti-goat IgG pAb (Life Technologies), then washed 3×, mounted in Prolong Gold with DAPI (Life Technologies), and imaged with a Zeiss LCM510 confocal microscope.

### Antigen presentation

Lung macrophages were infected or not with MuHV-4 (3 p.f.u./cell, 4h), washed, then incubated (18h, 37°C) in complete medium with 49100.2 T cells, which recognize an immunodominant, H2D^b^-restricted MuHV-4 epitope and express β-galactosidase (β-gal) from an NFAT-responsive promoter [58]. To assay β-gal the cells were washed in PBS and lysed in PBS / 5mM MgCl_2_ / 1% NP-40 / 0.15μM chlorophenol-red-beta-D-galactoside (CPRG, Merck Biosciences). 595nm absorbance was read after 2–4h.

### ELISA

MuHV-4 virions in 0.05% Triton-X100 / 50mM sodium carbonate pH = 8.5, were coated (18h, 4°C) onto Maxisorp ELISA plates (Nalge Nunc). The plates were washed x3 in PBS / 0.1% Tween-20, blocked with 1% bovine serum albumin / PBS / 0.1% Tween-20, incubated with 3-fold serum dilutions (1h, 23°C), washed x4 in PBS / 0.1% Tween-20, incubated (1h, 23°C) with alkaline phosphatase-conjugated goat anti-mouse IgG-Fc pAb (Sigma Chemical Co.), washed x5, and developed with nitrophenylphosphate substrate (Sigma). Absorbance was read at 405nm (Biorad).

## Supporting information

S1 Fig Lung infection shows an MHC II-dependent shift in MuHV-4 tropism. Single channel stains of MHC II-deficient mice (IA^-/-^) mice given MuHV-4 i.n. show a shift in lytic antigen staining (MHV) from type 1 alveolar epithelial cells at day 5 (co-distribution mainly with podoplanin (PDP)) to myeloid cells at day 9 (co-distribution mainly with CD68). I.n.-infected immunocompetent controls (IA^+/-^) show instead a general reduction in MHV staining from day 5 to day 9 in staining, without a change in distribution. See also Fig 1C.
(PDF)

S2 Fig Cell type-specific marker and MuHV-4 antigen staining in naive lungs. Naive mouse lungs were stained for podoplanin (PDP) to identify type 1 alveolar epithelial cells, and for CD68 to identify alveolar macrophages. Nuclei were stained with DAPI. MuHV-4 lytic antigen staining (MHV) was negative for both cell types.
(PDF)

S3 Fig MuHV-4 lytic antigen expression in macrophages of long-term infected IA^-/-^ mice. Single channel fluorescence signals are shown for 3 example lung sections of MHC II-deficient (IA^-/-^) mice, stained at 30 days after i.n. MuHV-4 for viral lytic antigens (MHV) and for myeloid cells (CD68). See also Fig 1D.
(PDF)

S4 Fig T cell depletion efficacy. **a.** Naive mice were given mAbs (i.p. 200μg x2) to CD4 (αCD4, GK.1.5), CD8 (αCD8α, 2.43), both (αCD4/8) or neither (control). 2 days later spleens were analysed for CD4^+^ and CD8^+^ T cells by flow cytometry using fluorochrome-conjugated mAbs H35-17.2 (αCD8β) and RM4-4 (αCD4, non-overlapping with GK1.5). Depletion from the gates shown was >95%.
**b.** Mice given mAbs as in **a** were infected i.n. with MuHV-4 (10^4^ p.f.u.). 10 days later sera were analysed for MuHV-4-specific IgG by ELISA. Each point shows the mean absorbance for samples from 3 mice. The lack of IgG response in αCD4 mice provided functional evidence of effective depletion.
(PDF)
