# Supplementary figures and images for: CD8+ T cell evasion mandates CD4+ T cell control of chronic gamma-herpesvirus infection
Source: PLoS Pathog. 2017 Apr 10;13(4):e1006311. doi: 10.1371/journal.ppat.1006311 (PMC5398720; doi:10.1371/journal.ppat.1006311)

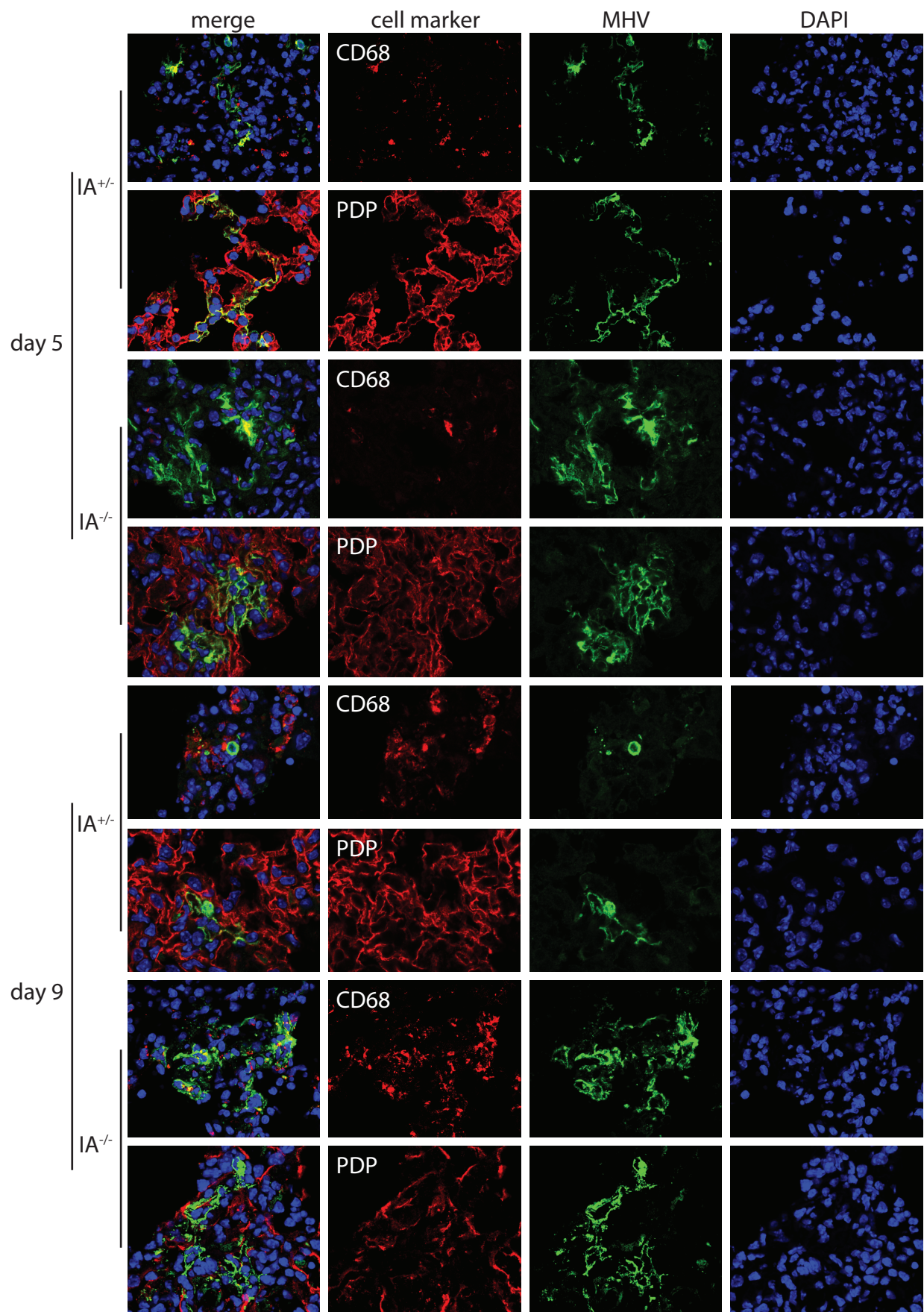

Supplement: S1 Fig — Single channel stains of MHC II-deficient mice (IA-/-) mice given MuHV-4 i.n. show a shift in lytic antigen staining (MHV) from type 1 alveolar epithelial cells at day 5 (co-distribution mainly with podoplanin (PDP)) to myeloid cells at day 9 (co-distribution mainly with CD68). I.n.-infected immunocompetent controls (IA+/-) show instead a general reduction in MHV staining from day 5 to day 9 in staining, without a change in distribution. See also Fig 1C. (PDF) [file ppat.1006311.s001.pdf]

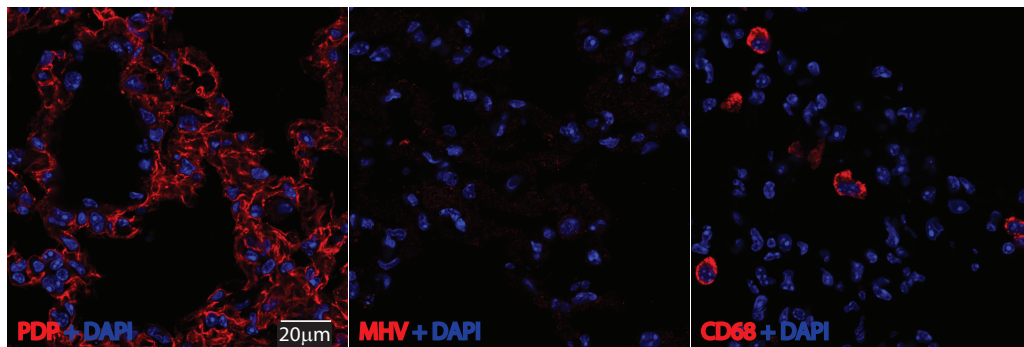

Supplement: S2 Fig — Naive mouse lungs were stained for podoplanin (PDP) to identify type 1 alveolar epithelial cells, and for CD68 to identify alveolar macrophages. Nuclei were stained with DAPI. MuHV-4 lytic antigen staining (MHV) was negative for both cell types. (PDF) [file ppat.1006311.s002.pdf]

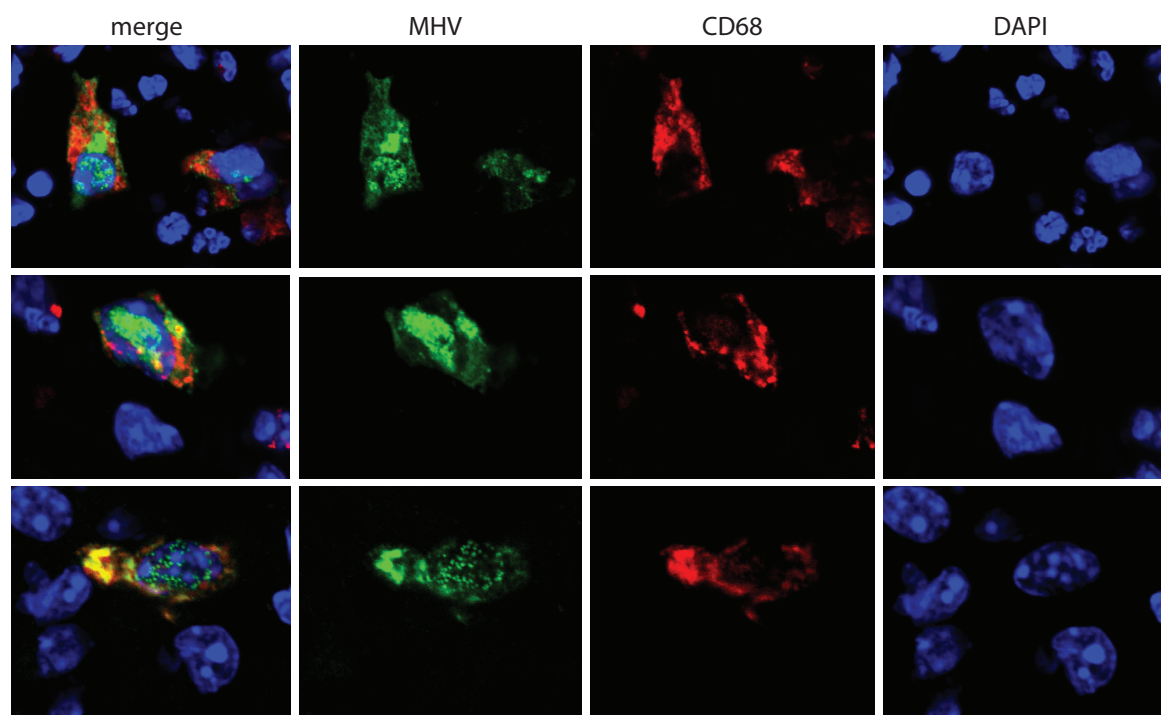

Supplement: S3 Fig — Single channel fluorescence signals are shown for 3 example lung sections of MHC II-deficient (IA-/-) mice, stained at 30 days after i.n. MuHV-4 for viral lytic antigens (MHV) and for myeloid cells (CD68). See also Fig 1D. (PDF) [file ppat.1006311.s003.pdf]

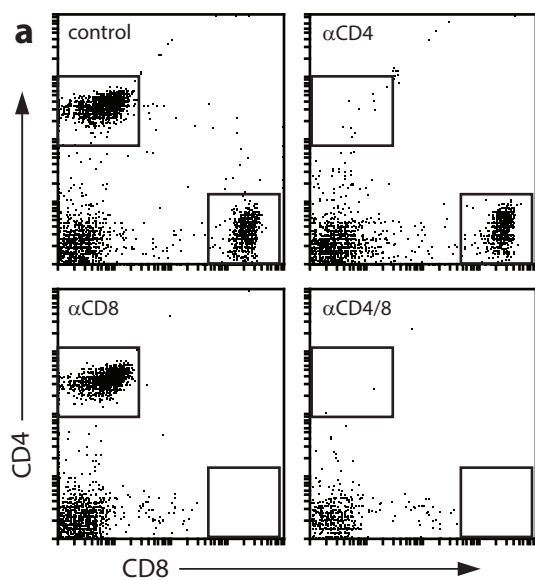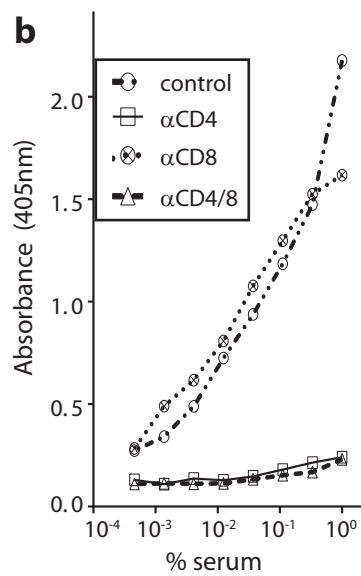

Supplement: S4 Fig — a. Naive mice were given mAbs (i.p. 200μg x2) to CD4 (αCD4, GK.1.5), CD8 (αCD8α, 2.43), both (αCD4/8) or neither (control). 2 days later spleens were analysed for CD4+ and CD8+ T cells by flow cytometry using fluorochrome-conjugated mAbs H35-17.2 (αCD8β) and RM4-4 (αCD4, non-overlapping with GK1.5). Depletion from the gates shown was >95%. b. Mice given mAbs as in a were infected i.n. with MuHV-4 (104 p.f.u.). 10 days later sera were analysed for MuHV-4-specific IgG by ELISA. Each point shows the mean absorbance for samples from 3 mice. The lack of IgG response in αCD4 mice provided functional evidence of effective depletion. (PDF) [file ppat.1006311.s004.pdf]
